# Torque Curve Optimization of Ankle Push-Off in Walking Bipedal Robots Using Genetic Algorithm

**DOI:** 10.3390/s21103435

**Published:** 2021-05-14

**Authors:** Qiaoli Ji, Zhihui Qian, Lei Ren, Luquan Ren

**Affiliations:** 1 Key Laboratory of Bionic Engineering, Jilin University, Changchun 130022, China; jiql17@mails.jlu.edu.cn (Q.J.); lqren@jlu.edu.cn (L.R.); 2 School of Mechanical, Aerospace and Civil Engineering, University of Manchester, Manchester M13 9PL, UK

**Keywords:** ankle push-off, planar biped robot, walking speed, energy efficiency, genetic algorithm, polynomial curve

## Abstract

Ankle push-off occurs when muscle–tendon units about the ankle joint generate a burst of positive power at the end of stance phase in human walking. Ankle push-off mainly contributes to both leg swing and center of mass (CoM) acceleration. Humans use the amount of ankle push-off to induce speed changes. Thus, this study focuses on determining the faster walking speed and the lowest energy efficiency of biped robots by using ankle push-off. The real-time-space trajectory method is used to provide reference positions for the hip and knee joints. The torque curve during ankle push-off, composed of three quintic polynomial curves, is applied to the ankle joint. With the walking distance and the mechanical cost of transport (MCOT) as the optimization goals, the genetic algorithm (GA) is used to obtain the optimal torque curve during ankle push-off. The results show that the biped robot achieved a maximum speed of 1.3 m/s, and the ankle push-off occurs at 41.27−48.34% of the gait cycle. The MCOT of the bipedal robot corresponding to the high economy gait is 0.70, and the walking speed is 0.54 m/s. This study may further prompt the design of the ankle joint and identify the important implications of ankle push-off for biped robots.

## 1. Introduction

The feet of bipedal robots are typically designed as flat feet, point feet, and curved feet [1]. Fully actuated biped robots are usually designed as having flat feet with actuated ankle joints. The representative fully actuated biped robot, Honda’s ASIMO, can realize complex locomotion by applying the control theory known as the zero moment point (ZMP) [2,3], such as walking up and down stairs, and turning in any direction [4]. Although this class of bipeds have good robustness and a quasi-statically stable walking gait, their energy efficiency is about 10 times that of a walking human. In order to improve the energy efficiency of a biped robot, McGeer built a planar passive bipedal walker without actuation, which can walk stably down shallow slopes with a humanlike gait [5]. The feet of passive bipedal robots are typically designed as curved feet to reduce energy consumption during the step-to-step transition. The curved feet have also been designed for the underactuated biped robot called ERNIE, and the results show that a larger radius foot or a smaller radius foot, whose center of curvature is located forward of the shank, can achieve similar energetic benefits across a range of motion [6]. The larger foot radius can help to improve the ability to handle disturbance rejection, enlarge the basin of attraction for walking, and reduce the vertical CoM variation [7]. Ruina et al. also designed a 2D bipedal robot named Ranger, with inner and outer legs and curved feet. The ankle of the biped Ranger can actively push off, powered by electric motors with cable drive. Results show that the energy consumption of Ranger is very close to that of humans [8]. However, the remarkable economy of these walkers comes at the cost of a poor ability to achieve tasks other than walking at a fixed speed [9]. Thus, many studies have been conducted on dynamically bipedal robots with point feet. While it can reduce the degrees of freedom and the control complexity of the biped robot, the absence of actuated ankle joints also reduces leg inertia [10,11], which can be more efficient for walking and running. This class of bipeds includes RABBIT [11], MABEL [12,13], and ATRIAS1.0 [14], among others. This type of biped mainly performs work about the hip and knee joints, which varies from that of human walking. Studies have shown that during normal walking, at a healthy human gait, the work produced about the ankle joint during the push-off phase accounts for more than 85% of the energy change of the center of mass (CoM) [15,16]. Furthermore, the stance ankle and foot together approximate the rolling motion of a wheel, imparting energy efficiency to the human gait [17,18], which is also a factor contributing to the high energy efficiency of human walking. The ankle joint has been regarded as a major power generator for human gait.

Studies have been explored on achieving stable walking gait and improving energy efficiency by using ankle push-off in bipedal robots. Based on the passive bipedal robot, a semi-passive bipedal robot was designed and can walk on level ground by using ankle push-off instead of gravity [19,20]. However, these types of bipeds can only walk at a fixed speed and cannot climb stairs, turn, or run [7]. Hobbelen et al. designed a planar biped robot called Meta at the Delft University of Technology, with actuation in the hip and ankle joints, which could achieve different walking speeds by changing the amount of ankle push-off [21]. However, further walking speed increases are limited by the passive swing knee motion of the biped [21]. Kuo et al. also demonstrated that push-off before heel-strike is four times more energy-efficient than push-off after heel strike in biped simulation. The reason is that the collision loss at the heel strike associated with velocity re-direction is reduced during step-to-step transition [22]. However, the ankle push-off of Meta starts when the leading leg lands, and stops when the torque in the trailing ankle is zero, so its energy efficiency was not significantly improved [21]. The robustness of biped robots has also been studied by using ankle push-off. Kim et al. proposed that discrete control of ankle push-off improves balance and disturbance rejection in a three-dimensional simulation of bipedal walking [23]. Daniel et al. simulated the feature of ankle push-off by using passive spring during biped robot walking, and compared robot data with human walking data. The results show that the ankle push-off powers leg swing [24]. However, unlike actuated muscles, passive spring cannot provide a sufficient forward thrust by using ankle push-off. Therefore, the ankle angle is significantly different between biped robot walking and human walking. Geng et al. suggested that active ankle joints are essential for bipedal robots to achieve fast walking. During the stance phase, the thrust generated by the ankle push-off powers the body forward [25,26]. On the basis of these studies, both the timing and amount of ankle push-off exert significant effects on the walking speed and energy efficiency during human walking.

Thus, the mechanism by which ankle push-off contributes to human walking has yet to be determined. Lipfert et al. studied the mechanism of ankle push-off during human walking and suggested that ankle push-off should be subdivided into an alleviation phase in which the trailing leg is alleviated from supporting the body mass, and a launching phase in which the stored elastic energy about the ankle is released [27]. Zelik et al. proposed that ankle push-off could not only increase the speed of the swing leg, but also accelerate the motion of the center of mass of the body [16]. However, the effect of ankle push-off on the walking speed and the energy efficiency of bipedal robots has not been deeply studied. Therefore, this study mainly aims to determine the optimal torque curve for ankle push-off that allows bipedal robots to acquire the maximum walking speed and the minimum MCOT by using the genetic algorithm (GA).

This study is organized as follows. In Section 2, we describe the simulation model and the method of joint target trajectory. Section 3 presents the controller of the biped robot and the optimization method. The results of the simulation analysis of our robot model are summarized in Section 4. Finally, Section 5 and Section 6 present the discussion and conclusions.

## 2. Methods

### 2.1. Simulation Model

**Figure 1:**
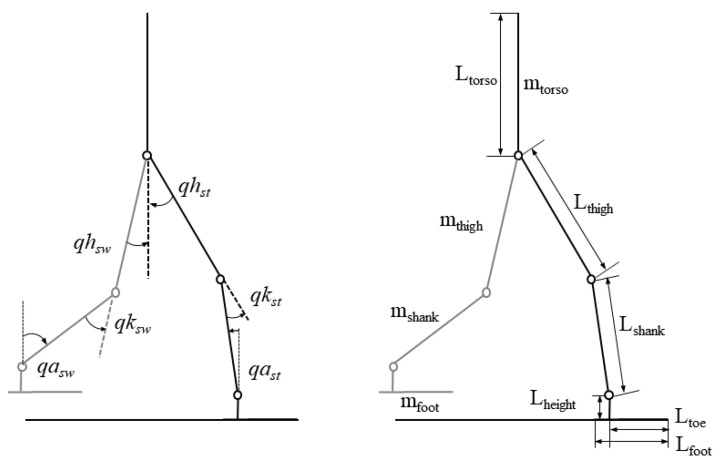
Simulation model of a bipedal robot.

The simulation analysis of the biped robot was conducted in the Simulink and Simscape Multibody toolbox in MATLAB R2020a. A planar biped robot model was developed with the actuation of the hip, knee, and ankle joints. As depicted in Figure 1, the biped walker model consisted of an upper body, two thighs, two shanks, and two feet. Each leg had three degrees of freedom. To further understand the effect of ankle push-off during human walking, the mechanical parameters of the simulated biped robot were based on human anthropometrics [23]. The mass of the simulated biped robot was about 74.2 kg and the leg length was 0.91 m. The hip width was 0.3 m and the foot length was 0.24 m. The specific parameters are shown in Figure 1 and Table 1.

In this study, the spatial contact force block has been used to simulate the interaction between robot foot and the ground. Four contact spheres were added to the four endpoints of the foot to construct a sphere-to-solid contact. The parameters of the contact model are shown in Table 1. The contact model consisted of two parts, including normal force and friction force. Normal force is defined by the contact spring stiffness and contact damping coefficient. Friction force consists of static friction and kinetic friction. Kinetic friction consists of Stribeck, Coulomb, and viscous forces, as shown in the literature [28]. The friction can be calculated by the Equation (1).

f = √2e (F_static_ − F_C_)∙exp(−(v/v_St_)^2^)∙v/v_St_ + F_C_∙tanh (v/v_Coul_) + F_V_·v (1)

v_St_ = v_static_√2 (2)

v_Coul_ = v_static_/10 (3)

where F_static_ is the static friction force, F_C_ is the Coulomb friction force, F_V_ is the viscous friction force, v_St_ is the Stribeck velocity threshold, v_Coul_ is the Coulomb velocity threshold, v_static_ is the breakaway friction velocity required for the foot to start sliding on the ground, and v is the velocity of the foot.

### 2.2. The Joint Trajectories

A complete gait cycle includes a stance period and a swing period in human walking. At the end of the stance phase, humans begin to push off prior to the heel strike of the leading leg, continuing positive work about the ankle even after heel strike and through most of the double support period [29,30]. When both legs touch the ground, that is, during the double support period, the control system of biped robots becomes complex. In this study, the biped robot only has a single support stage and instantaneous impact during the double support period [11]. With reference to the control method [11,31], when the leading leg strikes the ground, the trailing leg immediately lifts off the ground and begins to swing. The state machine is used to simulate the dynamic systems of a bipedal walking robot.

**Figure 2:**
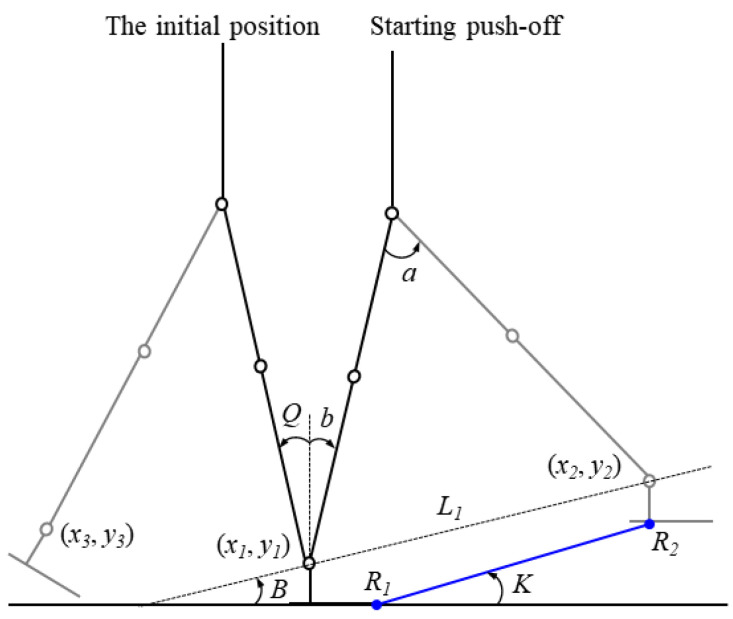
Initial position and push-off position. The black solid line indicates the stance leg, and the gray solid line indicates the swing leg.

Figure 2 shows the initial position and the push-off position. In this study, target positions of joints of the bipedal robot are calculated based on the global variable *Q.* The global variable *Q* is the angle of the stance leg with respect to the vertical line of the ground, as shown in Figure 2, and the variable *a* is the inter-leg angle. When *Q* is equal to *b*, and the inter-leg angel is equal to *a*, the biped posture is in the push-off position, and push-off begins. The distance between the ankle of the swing leg and the ankle of the stance leg is represented by *L*_1_. The angle *B* is measured between *L*_1_ and the horizontal plane. The toe position of the stance leg and the sole position of the swing leg are depicted by *R*_1_ and *R*_2_, respectively, and the angle with respect to the horizontal plane is *K*, which is defined as the push-off angle. Then, the calculation of the joint target trajectory is presented in detail. The real-time-space trajectory method consists of three steps. Firstly, we planned motion trajectories of the ankle joint during the swing phase, and the positions of the hip and knee joint of the swing and stance leg are calculated. Finally, the torque curve for the ankle composed of three-segment quintic polynomial curves is generated during the push-off phase.

#### 2.2.1. Ankle Positions of Swing Leg

The simulation analysis of the biped robot is conducted in the Simulink and Simscape Multibody toolbox in MATLAB R2020a. A measuring module, referred to as Transform Sensor in the Simscape toolbox was used to acquire the initial position of the ankle (*x*_1_, *y*_1_) of the stance leg and the ankle position of the swing leg (*x*_3_, *y*_3_), as shown in Figure 2. The ankle position (*x*_2_, *y*_2_) of the swing leg at the beginning of the push-off can be defined as follows:

*L*_1_ = 2*L*sin (a/2) (4)

B = 90° − (180° − a)/2 − b(5)

*x*_2_ = *x*_1_ + *L*_1_cos (B) (6)

*y*_2_ = *y*_1_ + *L*_1_sin (B) (7)

where *L* is the leg length from the hip joint to the ankle joint.

**Figure 3:**
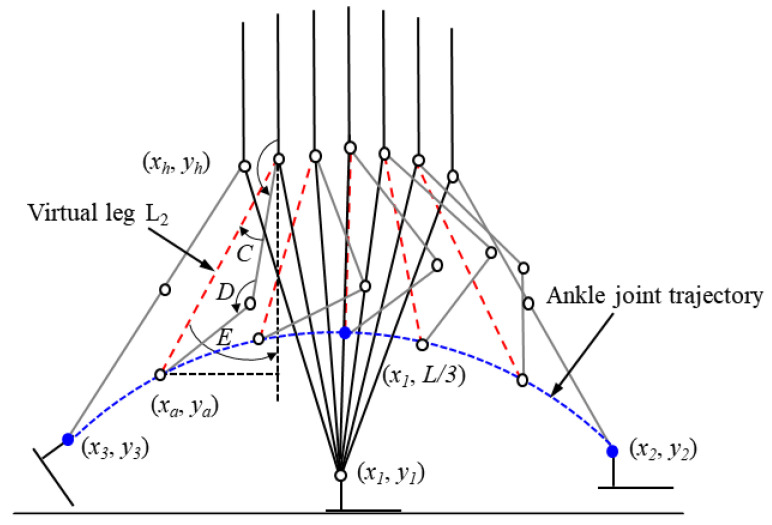
Hip and knee positions of the swing leg. The red dotted line indicates the virtual leg. The blue dotted line indicates the ankle joint trajectory of the swing leg.

To identify the ankle positions of the swing leg in advance, the position (*x*_1_, *L*/*3*) above the ankle joint of the stance leg is selected as the necessary point of the ankle trajectory of the swing leg. The starting (*x*_3_, *y*_3_), middle (*x*_1_, *L*/*3*), and ending positions (*x*_2_, *y*_2_) of the ankle of the swing leg are selected, and the quadratic polynomial is used to identify the ankle positions during the swing period, as shown in Figure 3. In MATLAB, the polyfit function is used to calculate the quadratic polynomial coefficient. The function of the ankle position during the swing period is determined by *f*(*x*), with *x* ranging from *x*_3_ to *x*_2_. The proportion of the global variable *Q* in the range of motion of the stance leg (*Q*_0_, *b*) is then determined using Equation (8). The corresponding horizontal and vertical coordinates of the ankle positions (*x_a_*, *y_a_*) are defined by Equations (9) and (10). The hip positions (*x_h_*, *y_h_*) of the swing leg are defined by Equation (8).

v = (*Q* − *Q*_0_)/(b − *Q*_0_) (8)

*x_a_* = *x*_3_ + (*x*_2_ − *x*_3_) v (9)

(*x_a_*, *y_a_*) = (*x_a,_* f(*x*_a_)) (10)

(*x_h_*, *y_h_*) = (*x_1_* − Lsin(Q), *y*_1_ + Lcos(*Q*)) (11)

where *Q*_0_ is the global variable when the leading leg just touches the ground during the early stance phase.

#### 2.2.2. Target Positions of Hip and Knee Joints

Stick diagrams of the bipedal robot changed from the initial position to the beginning position of push-off are shown in Figure 3. The hip and knee positions are denoted by *qh_sw_* and *qk_sw_* and defined by Equations (16) and (17).

*L*_2_ = norm (*x*_h_ − *x*_a_) (12)

E = acos ((*y*_h_ − *y*_a_)/*L*_2_)) (13)

C = acos ((*L*_2_^2^ + *L*_thigh_^2^ − *L*_shank_^2^)/2*L*_thigh_*L*_2_) (14)

D = acos ((*L*_thigh_^2^ + *L*_shank_^2^ − *L*_2_^2^)/2*L*_thigh_*L*_shank_) (15)

*qh_sw_* = 180° − (E − C) (16)

*qk_sw_* = 180° − D (17)

where *L*_2_ is the length of the virtual leg, C is the angle between the thigh of the swing leg and the virtual leg, D is the angle between the thigh and shank of the swing leg, and E is the angle between the virtual leg and the vertical line of the ground.

#### 2.2.3. Ankle Torque during Push-Off Phase

**Figure 4:**
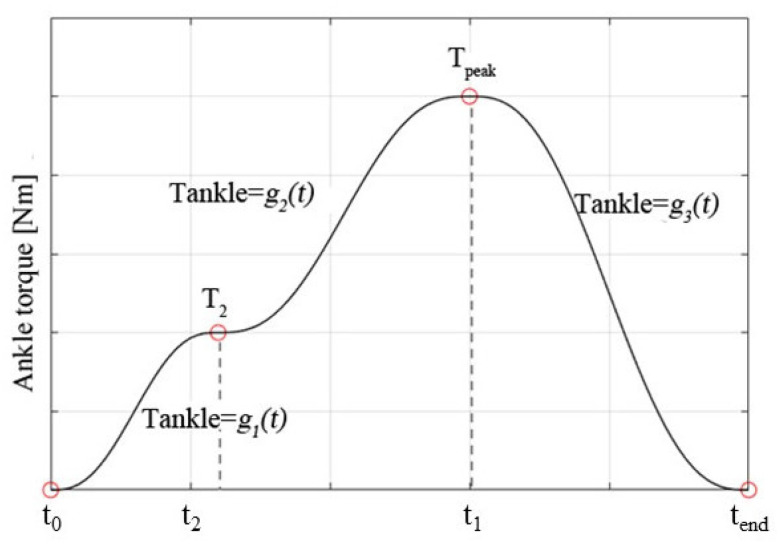
Ankle torque during the push-off phase.

**Figure 5:**
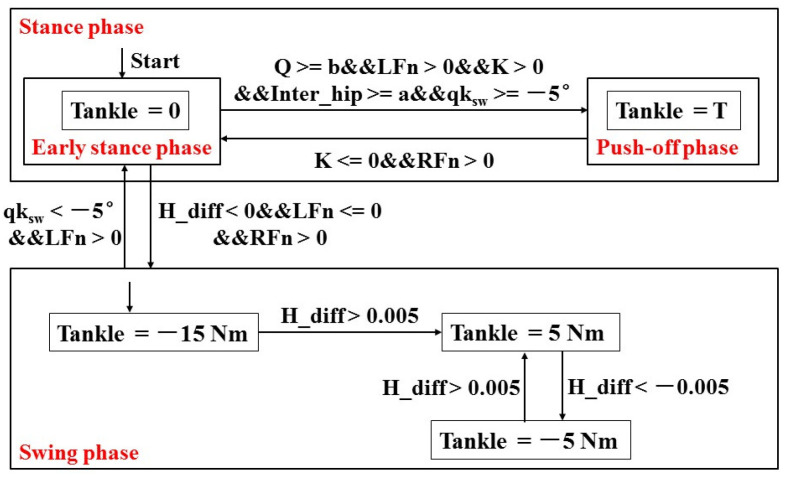
State machine of ankle torque. Tankle indicates ankle torque, and T indicates the ankle torque during the push-off phase. LFn and RFn represent ground reaction forces. Inter_hip denotes the angle between the two legs. H_diff is defined by the difference between the toe and the heel of the swing leg.

At the push-off stage, the torque curve formed by three-segment quintic polynomial curves is applied to the ankle of the stance leg according to the torque curve of the ankle joint during human walking. The torque curve for the ankle joint during push-off is presented in Figure 4. The main parameters include *t*_0_ and *t*_end_ at the start and at the end of the push-off phase, respectively, and the corresponding torque is 0. Transition timing is *t*_2_ with the corresponding ankle torque T_2_. Peak timing is *t*_1_ with the corresponding peak ankle torque T_peak_. The torque curves of the ankle are obtained by changing the range of peak timing *t*_1_, transition timing *t*_2_, peak torque T_peak_ and transition torque T_2_. When the stance feet lift off the ground, the stance leg begins to swing after the push-off stage. To prevent the foot from stumbling with the ground, the state machine of ankle torque is used to keep the bottom of the foot parallel with the ground during the swing phase, as shown in Figure 5. When the global variable *Q* is equal to the angle b, the biped robot starts to push-off. The push-off angle K is then defined by Equation (18).

K = atan((*y*_sole_ − *y*_toe_)/(*x*_sole_ − *x*_toe_)) (18)

where R_1_ (*x*_toe_, *y*_toe_) is the toe position of the stance leg, and R_2_ (*x*_sole_, *y*_sole_) is the sole position of the swing leg. The torque curve for the ankle joint during the push-off phase corresponds to the process when the push-off angle changes from K to 0°. To simplify the target trajectory planning, the horizontal axis (*t*_0_, *t*_end_*)* of the planned curve of ankle torque is set to range from 0% to 100%. The push-off angle *K* then corresponds linearly to the planned ankle torque.

## 3. Controller

### 3.1. Controller Framework of the Ankle Joint

The controller framework of the ankle joint during the single gait cycle is presented in Figure 5. The ankle torque of the biped robot during the single gait cycle mainly includes two stages: the stance stage and the swing stage. The stance stage includes the early stance stage and the push-off stage. When the stance leg is at the early stance stage and the ankle joint is in the passive mode, the ankle torque is 0. As the swing leg moves forward to the initial position of push-off, the ankle joint of the stance leg starts to push-off. The initial position is the state just after the push-off phase; thus, the stance leg is at the early stance phase, and the corresponding ankle torque is 0 N∙m. As the stance leg moves forward, when the global variable *Q* is greater than or equal to the angle b, the inter-leg angle is greater than or equal to a, and the knee angle qk_sw_ of the swing leg is greater than −5°, the left leg begins to push-off. The torque curve is applied to the ankle joint of the left leg. When the push-off angle K is less than or equal to 0°, and the ground reaction force of the right leg is greater than 0 N, the ankle torque becomes 0 N∙m at the end of the push-off phase. When the height difference between the toe and heel of the left foot (H_diff) is less than 0, the ground reaction force of the left leg (LFn) is less than or equal to 0 N, the ground reaction force of left leg (RFn) is greater than 0 N, and the left foot lifts off the ground and begins to swing. To prevent the foot from stumbling with the ground, the height difference is considered as the transition of the state machine of the swing stage. The height difference of the left foot is used to control the ankle angle so that the sole of the foot is basically parallel to the ground.

### 3.2. Overall Framework of the Bipedal Robot

**Figure 6:**
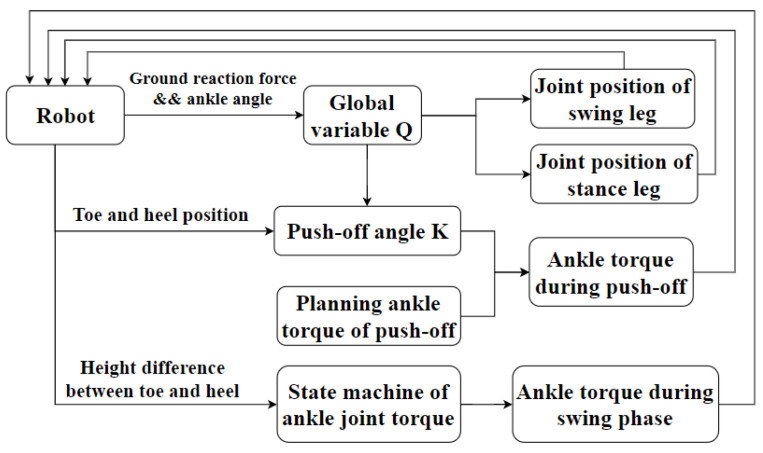
Overall controller framework of the planar biped robot.

The overall controller framework of the bipedal robot is shown in Figure 6. The global variable *Q* is determined by the ground reaction force and the ankle angle qa_st_. The target positions of the hip and knee joints of both legs are calculated using the global variable *Q*. When the posture of the bipedal robot is consistent with the push-off position, and the global variable *Q* is equal to b, the ankle torque of the stance foot is calculated based on the push-off angle K during the push-off phase. The proportional-derivative (PD) controller is used to command the desired position of the hip and knee joints. The amount of the ankle push-off is applied to the ankle joint of the stance leg. The ankle joint is actuated by the torque mode in the Simscape toolbox. Ankle torque during the swing phase is calculated using the height difference between the toe and the heel of the swing leg on the basis of the state machine of the ankle joint torque, as shown in Figure 5.

### 3.3. Optimization Method

The walking speed can be controlled by the amount of ankle push-off [9]. The timing of ankle push-off is particularly important for energy-saving mechanisms and walking speed. The genetic algorithm (GA) is a simple and powerful search method that proved to be effective to find the global optimal solution and avoid being trapped in a local optimal solution like with traditional methods. In this study, the GA has been used to find the optimal torque curve of push-off and to determine the fast walking speed of the bipedal robot. The walking distance and the mechanical cost of transport (MCOT) are selected as the objective function. MCOT is defined as the amount of energy that the bipedal robot uses per distance traveled per weight of the walker [21]. The smaller the MCOT, the more energy-efficient of bipedal robot [9]. The running time of the simulation model is 30 s [24]. The longer the walking distance, the higher the walking speed of the bipedal robot. The walking speed is calculated using the ratio of the walking distance to the total motion time of the biped robot. When the robot completes 30 s of normal walking, MCOT is calculated using Equation (19), otherwise, MCOT is 10. the objective function is defined by Equations (20) and (21).

(19) $$\mathrm{MCOT}=\frac{\mathrm{Used}\;\mathrm{energy}\;}{\mathrm{Weight}\times \mathrm{Distance}\;\mathrm{traveled}}=\frac{\sum_{\mathrm{all}\;\mathrm{joints}}\int_{0}^{T_{t}}\left|\tau \cdot \dot{\varphi }\right|\cdot d_{t}}{m\cdot g\cdot s}$$

(20) $$f_{1}=-s$$

(21) $$f_{2}\left(T\right)=\{\begin{array}{ll} 10, & T<30\\ \mathrm{MCOT}, & T\geq 30 \end{array}$$

where T_t_ is the movement time of the biped robot, τ is the joint torque (N∙m), $\dot{\varphi }$ is the joint angular velocity (rad/s), m is the robot mass, s is the walking distance, and g is gravity.

The optimization parameters of the torque curve for the ankle are the timing of the peak torque *t*_1_, the transition time *t*_2_, the transition torque T_2_, the push-off timing determined by the angle b, and the inter-leg angle a. For the convenience of parameter optimization, the two ratios Ratio_1_ (*t*_2_/*t*_1_) and Ratio_2_ (T_2_/T_peak_) are defined to determine the proportion between the transition point and the peak point. Because the parameters to be optimized in the genetic algorithm (GA) need to be integer, we defined the Ratio_1_* and Ratio_2_*. The Ratio_1_* is equal to the produce of Ratio_1_ and coefficient 10. The Ratio_2_* is equal to the produce of Ratio_2_ and coefficient 10. The ranges of the optimization parameters are listed in Table 2. The parameters to be optimized can be expressed as the vector *p* = [*t*_1_, Ratio_1_*, Ratio_2_*, b, a].

The value of the initial population is 35, and the genetic generation is 50 in the optimization analysis. According to the ankle torque of humans during the push-off phase, the peak torque is selected as 172 N∙m. To accelerate the optimization speed of the simulation model, when one of the three stopping criteria is satisfied, the simulation ends immediately. The walking distance of the bipedal robot does not change, and the duration is longer than 1 s. The ground reaction forces of both feet are 0, and the duration is greater than 0.1 s. The height of the torso is less than 0.6 m or greater than 1.2 m.

## 4. Results

### 4.1. Optimal Results

**Figure 7:**
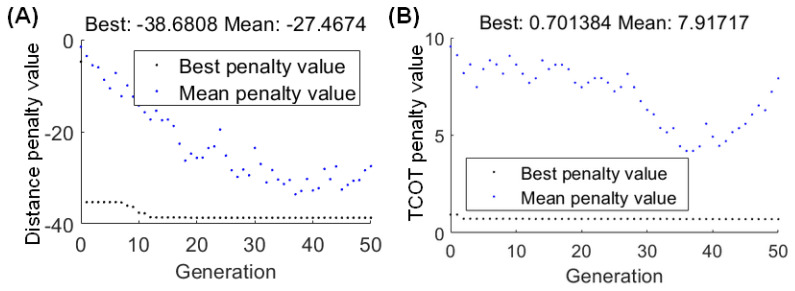
Optimal results of the genetic algorithm. (**A**) Walking distance. (**B**) Mechanical cost of transport.

When the optimal parameters of the torque curve for the ankle joint are *t*_1_ = 8, Ratio_1_* = 2, Ratio_2_* = 8, b = 15°, and a = 53° determined using GA, the longest walking distance of the bipedal robot is 38.68 m, and the maximum walking speed achieved by the robot is 1.30 m/s, as shown in Figure 7A. The Froude number, Fr, is used to describe the dynamical similarity of legged locomotion over a wide range of animal sizes and speeds [30]. The normal value of humans’ Fr is about 0.20 during normal walking. The Fr of the bipedal robot is 0.23, calculated using Equation (22), which is close to that of humans. Thus, the simulated bipedal robot has a human-like walking gait. According to the literature [31], the normal walking speed of humans is 1.32 m/s, and the step length and step frequency are 1.13 m and 1.18 Hz. The step length and step frequency of the simulated robot are 0.77 m and 1.67 Hz. The step frequency of simulated robot is larger than that of humans. However, the step length of simulated robot is smaller than that of humans at the almost equal walking speed.

Fr = v^2^/g (22)

where v is the walking speed, g is gravity, and l is the leg length.

The minimum MCOT of the bipedal robot is 0.70 by using GA, as shown in Figure 7B, and the corresponding parameter combinations are *t*_1_ = 4, Ratio_1_* = 8, Ratio_2_* = 6, b = 15°, and a = 55°. At this point, the walking speed of the robot is 0.54 m/s. Studies show that when the human walking speed is equal to 0.54 m/s, the corresponding MCOT is about 0.45 [32].

### 4.2. Joints Kinematics at Different Speeds

**Figure 8:**
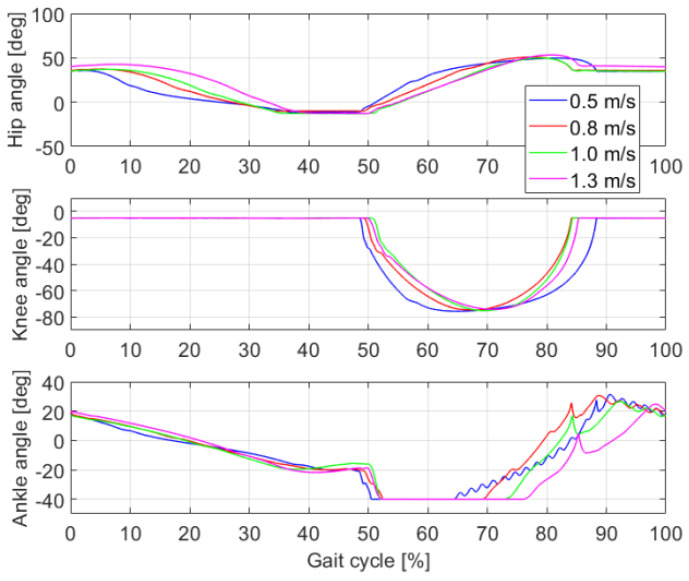
Joints kinematics of the simulated bipedal robot.

To further study the influence of ankle push-off on the walking speed of bipedal robots, the joints kinematics during one gait cycle of the bipedal robot at four walking speeds are identified, as shown in Figure 8. The optimal parameters of the torque curve for the ankle joint corresponding to the four walking speeds are listed in Table 3. When the walking speed changes from 0.5 m/s to 1.3 m/s, the hip, knee, and ankle joints of the bipedal robot exhibit similar movement trends. At the early stance phase, the hip joint flexes from 40° to −10°, the knee joint remains at −5°, and the ankle angle is reduced from 20° to −20°. During the ankle push-off phase, the ankle angle increases, whereas the hip and knee remain stable. During the swing period, the hip joint is extended from −10° to 50° and is then decreased to 40°. The knee joint is firstly flexed to −75° and then extended to −5°. To prevent the foot of the swinging leg from stumbling with the ground, the state machine of the ankle torque is used to keep the foot parallel to the ground. Thus, the ankle angle fluctuates slightly during the swing phase.

### 4.3. Torque and Power of the Ankle Joint

**Figure 9:**
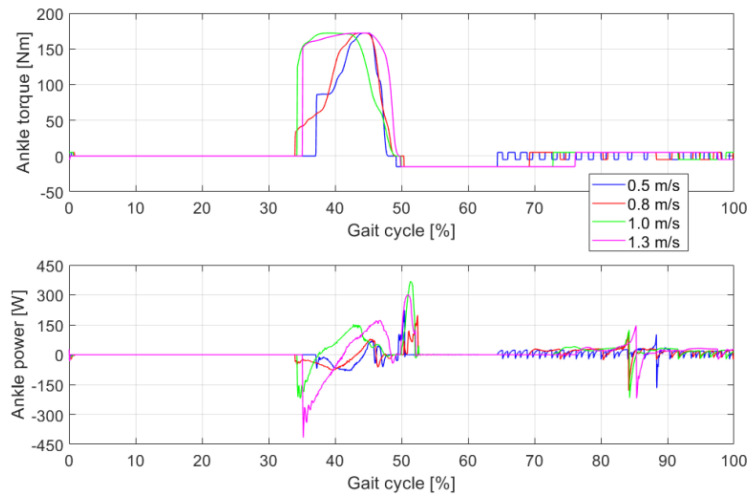
Ankle torque and power during one gait cycle at different walking speeds.

Figure 9 shows the ankle torque and power of the bipedal robot under different walking speeds during one gait cycle. At the early stance, the ankle joint is in a passive state, the ankle torque is 0 and the output power of the ankle is also 0. When the planned torque curve is applied to the ankle joint, the ankle first executes negative work. The body weight remains supported by the stance leg at the early stance. As the trailing leg moves forward around the ankle, the ankle angle decreases, and the angular velocity is negative. When the body weight is transferred to the front of the trailing leg, the ankle begins to execute positive work. Ankle power with a positive value indicates that the bipedal robot starts to push off. Ankle power with a negative value indicates that the push-off of the bipedal robot has ended. The ankle push-off of the bipedal robot occurs longer at a fast speed (1.3 m/s) than at a slow speed (0.5 m/s). The negative and positive work generated about the ankle joint during fast walking is significantly greater than that generated at a low speed.

**Figure 10:**
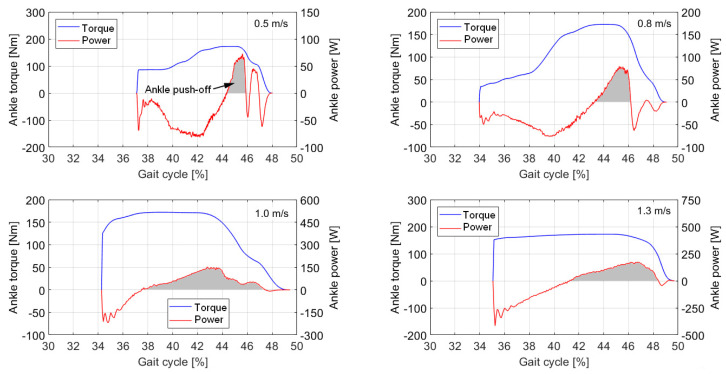
Ankle torque and power during the push-off phase at different walking speeds. The blue line indicates ankle torque. The red line indicates ankle power. The gray area represents the mechanical work of ankle push-off, when the ankle power is positive.

Figure 10 shows the push-off torque and power produced by the ankle joint of the bipedal robot at different walking speeds. The gray area represents the positive work produced by the ankle joint during the ankle push-off phase. When the walking speed is 0.5 m/s, the ankle torque occurs at approximately 37.1% of the gait cycle. When the walking speeds are 0.8 m/s, 1.0 m/s, and 1.3 m/s, the ankle torque curve occurs at approximately 34% of the gait cycle. With increasing speed, the timing of the ankle torque occurs earlier. When the speed increases to a certain extent, the timing difference of the ankle torque is small. When the walking speed is 0.5 m/s, the timing of the ankle push-off occurs at 44.4% of the gait cycle. When the walking speed is 0.8 m/s, the timing of ankle push-off occurs at 43.3% of the gait cycle. When the speed is 1.0 m/s, the timing of ankle push-off occurs at 37.5% of the gait cycle. With increasing walking speed, the timing of ankle push-off occurs earlier, and the timing difference is significant. However, when the speed is 1.3 m/s, the timing of ankle push-off occurs at 41.3% of the gait cycle, which may be due to the increasing negative work generated by the ankle joint.

### 4.4. Walking Gaits

**Figure 11:**
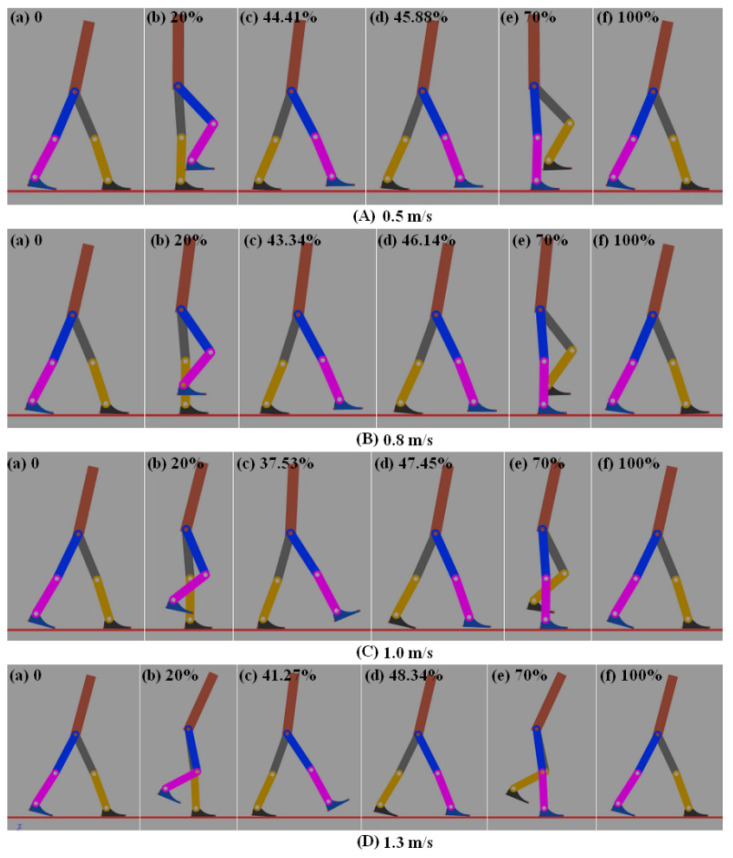
Snapshots of the simulated bipedal robot. (**A**) 0.5 m/s (**B**) 0.8 m/s (**C**) 1.0 m/s (**D**) 1.3 m/s.

The one gait cycle is defined from the leg touch-down on the ground to the subsequent touch-down of the same leg. Snapshots of the bipedal walking robot during the gait cycle are shown in Figure 11. The left leg of the bipedal robot just touches the ground, as shown in Figure 11A(a). In Figure 11A(c), the push-off of the bipedal robot begins, and in Figure 11D(d) the ankle push-off ends. When the walking speed is 0.5 m/s, the ankle push-off occurs at 44.41–45.88% of the gait cycle. The start point of ankle push-off begins at 44.41% of gait cycle, when the ankle power becomes positive. with a duration of 1.47%, as shown in Figure 11A(c,d). When the walking speed is 0.8 m/s, the ankle push-off occurs at 43.34–46.14% of the gait cycle, with a duration of 2.80%, as shown in Figure 11B(c,d). When the walking speed is 1.0 m/s, the ankle push-off occurs at 37.53–47.45% of the gait cycle, with a duration of 9.92%, as shown in Figure 11C(c,d). When the walking speed is 1.3 m/s, the ankle push-off occurs at 41.27–48.34% of the gait cycle, with a duration of 7.06%, as shown in Figure 11D(c,d). In addition, the torso inclination rises with increasing speed during 20% and 70% of the gait cycle, as shown in Figure 11B(b,e).

### 4.5. Mechanical Work of Joins

**Figure 12:**
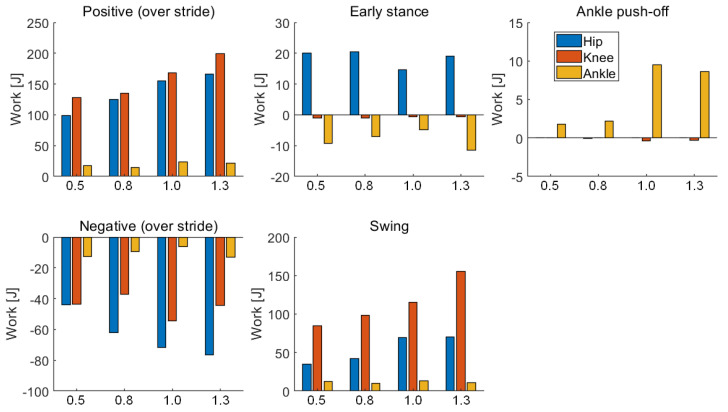
Mechanical work of the joints at different walking speeds. Positive and negative work during the gait cycle, including the early stance, ankle push-off, and swing phase.

The positive and negative work generated by the hip, knee, and ankle of the bipedal robot during one gait cycle at different walking speeds are presented in Figure 12 and Table 4. The mechanical work performed by the joint of the biped robot is the integral over time of the mechanical power, which is defined by the product of the joint torque and joint velocity. With increasing walking speed, the positive work produced by the hip and the knee increases during one gait cycle, whereas the positive work produced by the ankle shows no significant change. The knee joint produced the most positive work and the hip joint produced the most negative work during the gait cycle at different walking speeds. The positive work of the knee joint at the fast walking speed (approximately 199.03 J at 1.3 m/s) is 56.05% larger than that at the low walking speed (approximately 127.54 J at 0.5 m/s). The negative work of the hip joint at the fast walking speed (approximately −76.42 J at 1.3 m/s) is 73.80% larger than that at the low walking speed (approximately −43.79 J at 0.5 m/s).

The whole gait cycle can be divided into three phases: the early stance phase, ankle push-off, and the swing phase. In the early stance, the hip joint mainly generates positive work, whereas the knee and ankle joints produced negative work. The hip joint contributes to push the center of mass forward and keep the torso leaning forward. The ankle joint mainly contributes to the positive work during the ankle push-off phase and provides energy for the bipedal robot system. The ankle push-off may reduce the velocity of the impact of the leading leg and pushes upward on the center of mass. The work generated by the hip and knee joints comprises only a small portion of total energy, which means that the hip and knee joints are almost passive state during ankle push-off phase. At a fast walking speed (approximately 8.69 J at 1.3 m/s), the work produced by the ankle during ankle push-off is 4.82 times than that produced at a low walking speed (approximately 1.79 J at 0.5 m/s). With increasing walking speed, the work produced by the ankle joint during ankle push-off phase firstly increases and then decreases. During the swing phase, the work generated by the knee and hip joints gradually increase with increasing speed. The work generated by the knee joint at the fast walking speed (approximately 155.45 J at 1.3 m/s) is 83.27% larger than that at the slow walking speed (approximately 84.82 J at 0.5 m/s). The work produced by the knee joint contributes to flex the swing leg and prevents the foot of the swing leg from stumbling on the ground. In the early stance, the work produced by the hip joint during fast walking (at speeds of 1.0 m/s and 1.3 m/s) is significantly less than that generated during walking at the low speeds (at speeds of 0.5 m/s and 0.8 m/s). While the work generated by the ankle joint during the ankle push-off phase during fast walking is significantly greater than that generated at a low speed.

### 4.6. Energy Efficiency

**Figure 13:**
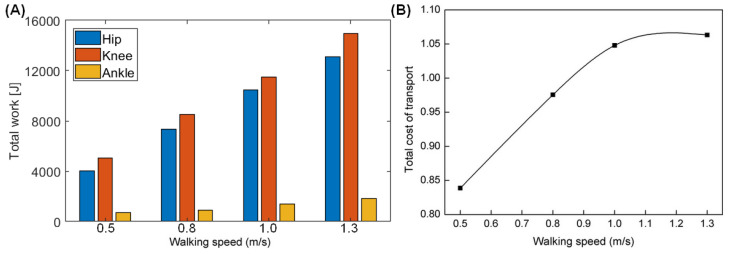
Energy use varies with the walking speed. (**A**) Total mechanical work; (**B**) MCOT.

Figure 13 shows the total change in energy of the bipedal robot during walking. The mechanical work of the hip, knee, and ankle varies with speed, as shown in Figure 13A, and the MCOT of the bipedal robot changes with speed as shown in Figure 13B. The work produced by each joint of the robot increases with increasing speed, with most of the work produced by the knee and while the least produced by the ankle joint. The energy consumption of the robot increases with increasing speed, because more work is needed to change the motion direction of the center of mass during step-to-step transition [21]. The maximum walking speed of the biped robot is 1.3 m/s, with a corresponding MCOT of about 1.06. Studies have shown that during normal walking, human beings choose the optimal gait corresponding to the lowest energy consumption. The preferred walking speed is 1.21 m/s, and the corresponding MCOT is 0.33 [32,33]. By comparison, the energy consumption of the biped robot (at speed of 1.3 m/s) is about 3.2 times that of human beings (at speed of 1.21 m/s).

## 5. Discussion

Hobbelen et al. proposed that the walking speed of the bipedal robot Meta can be changed by adjusting ankle push-off [21]. The maximum walking speed of the simulated bipedal robot (1.3 m/s, Fr = 0.43) is larger than that of Meta (0.68 m/s, Fr = 0.28). Further walking speed increases of Meta are limited by the passive knee joint. However, the ankle push-off of Meta occurs after the leading leg lands. Studies show that push-off timing considerably affects energy efficiency. The push-off before heel-strike is four times more energy-efficient than that after heel-strike [27]. Thus, the energy efficiency of Meta has not been improved significantly by ankle push-off. This study aims to explore whether the push off timing affects the walking speed under the condition that the peak torque of the ankle is constant. The only energetic loss is incurred during the collision of the swing leg with the ground [27]. The concentrated force generated by the ankle push-off pushes upward on the center of mass and reduces the velocity of the impact and energy loss. Therefore, appropriate push-off timing is significant for determining whether the biped robot can achieve a higher walking speed under low energy consumption. In this study, when the walking speed is 1.0 m/s, push-off timing occurs at 37.53% of the gait cycle, and when the walking speed is 1.3 m/s, push-off timing occurs at 41.27% of the gait cycle. The work generated by the ankle joint during ankle push-off phase is greater at a low speed than that at a fast speed, as shown in Figure 12, indicating that an appropriate push off timing is significant for trade-offs between energy efficiency and walking speed. 

About 80% of the change in push-off limb energy contributes directly to the CoM energy changed by ankle push-off during human walking [15]. In addition, the soft plantar tissue and the elasticity of the Achilles tendon can recover part of the energy during push-off [31]. Thus, humans can perform tasks with high energy efficiency [21]. Reduced ankle push-off contributes to increased metabolic energy expenditure during human normal walking. In the current study, the work generated by the ankle push-off during walking at a high speed (at speeds of 1.0 m/s and 1.3 m/s) is significantly higher than that during walking at a low speed (at speeds of 0.5 m/s and 0.8 m/s), whereas the work produced by the hip joint decreases. Therefore, the ankle push-off reduces the work generated by the hip joint and the overall energy consumption of the bipedal robot. Moreover, the bipedal robot has no double support phase. However, a double support phase accounts for 20% of the gait cycle in normal human walking [7]. Humans begin push-off prior to heel strike of the leading leg, and positive work about the ankle continues even after heel strike and through most of the double support period [27]. When the walking speed of human is 1.25 m/s, ankle push-off occurs at 50–65% of the gait cycle [31]. When the walking speed of the bipedal robot is 1.3 m/s, ankle push-off occurs at 41–48% of the gait cycle. Compared with those of humans, the push-off timing of the bipedal robot occurs earlier, and the push-off duration of the bipedal robot is shorter. Moreover, the ankle joint of the bipedal robot is in the passive state at the early stance. When ankle torque is applied, negative work is generated before the robot starts producing positive work and enters the push-off phase. This finding also varies from the increase in ankle torque during human walking in the early stance phase, which may be a factor impeding the improvement of the walking speed of the bipedal robot. 

When its walking speed is close to that of humans, the simulated bipedal robot has an energy consumption of approximately 3.2 times that of humans. This result may be attributable to the deformation of soft tissue, such as muscles, tendons, plantar fascia, cartilage, etc., which contributes to energy dissipation and return. Finally, the deformation of soft tissue helps to improve energy efficiency [31]. However, the simulated bipedal robot is composed of a rigid structure without elastic energy storage elements, potentially leading to their high energy consumption. The MCOT of the simulated biped robot is 0.7. The MCOT of humans is 0.05 [34]. Humans rely heavily on passive limb dynamics and power their walking gait predominantly with ankle push-off. Additionally, muscle total efficiency is about 25%, comparable to the 23% delivered to a vehicle driven train by an engine [27]. The muscle-tendon unit can also restore and release energy during walking. In this study, the simulated biped robot only had rigid links, without any elastic elements. Moreover, the hip and knee joints of the simulated robot are actively controlled during walking without passive dynamics. Therefore, the MCOT of simulated robots is much higher than that of humans.

Finally, bipedal robots can rely on two powering strategies for dynamic walking. One powering strategy is to produce torque by the hip joint, and the other is to push off with the ankle of the trailing leg [27]. The bipedal robots may need the actuation combination of the hip and ankle to strike a balance between energy consumption and walking speed. The hip joint produces a small amount of work only when necessary, and ankle push-off provides the main energy for a change in CoM energy. Ankle push-off can also accelerate the movement of the swinging leg and improve the walking speed of the bipedal robot. The appropriate push-off timing is crucial to energy efficiency. Therefore, the cooperation between effective ankle push-off and the elastic ankle structure may increase walking speed and energy efficiency. In addition, the wind and other environmental factors have not been considered during the simulation.

## 6. Conclusions

In this study, the global variable *Q* between the stance leg and the vertical line of the ground is used to determine target positions of the hip and knee joints of both legs. When the global variable *Q* is equal to b, the bipedal robot starts to push off, and the torque curve for the push-off is applied to the ankle joint on the basis of the push-off angle K. With the walking distance and the mechanical cost of transport (MCOT) as the objective functions, the genetic algorithm (GA) is used to obtain the optimal torque curve for the ankle push-off. The results indicate that the maximum walking speed obtained by the bipedal robot is 1.3 m/s, and the ankle push-off occurs at 41.27–48.34% of the gait cycle. The minimum MCOT of the bipedal robot corresponding to the high economy gait is 0.70, and the corresponding walking speed is 0.54 m/s. This study may encourage the ankle design of bipedal robots and identify the important implications of ankle push-off for bipedal robots.

## Figures and Tables

**Table 1:** Model parameters.

Parameters	Mass/kg	Length/m	Parameters	Values
Torso	54.2	0.66	Coefficient of contact stiffness (N/m)	75,000
Thigh	6.6	0.45	Coefficient of contact damping (N/(m/s))	4600
Shank	2.7	0.37	Coefficient of static friction	0.8
Foot	0.8	0.24	Coefficient of kinetic friction	0.6
Foot—toe	-	0.192	Contact sphere radius(m)	0.01
Foot—height	-	0.09		

**Table 2:** Limits of optimization variables.

Parameters	Lower Limit	Upper Limit
*t*_1_	2	8
Ratio_1_*	2	8
Ratio_2_*	2	8
b/°	10	20
a/°	40	60

**Table 3:** Optimized variables of the torque curve for the ankle joint at different walking speeds.

Peak Time *t*_1_	Ratio_1_*	Ratio_2_*	b/°	a/°	Walking Speed (m/s)
7	7	5	13	46	0.5
7	5	2	12	46	0.8
5	2	7	15	48	1.0
8	2	8	15	53	1.3

**Table 4:** Joint mechanical work.

Mechanical Work (J)	Walking Speed (m/s)	Positive (Stride)	Negative (Stride)	Net (Stride)	Early Stance Phase	Ankle Push-Off Phase	Swing Phase
Hip	0.5	99.30	−43.97	55.33	20.11	0.05	35.27
	0.8	124.77	−62.14	62.63	20.38	−0.03	42.28
	1.0	155.44	−71.69	83.75	14.58	−0.01	69.19
	1.3	166.16	−76.42	89.74	19.06	0.05	70.64
Knee	0.5	127.54	−43.75	83.79	−1.03	0.00	84.82
	0.8	134.67	−37.30	97.37	−1.05	0.00	98.42
	1.0	168.35	−54.43	113.92	−0.68	−0.39	114.98
	1.3	199.03	−44.54	154.49	−0.67	−0.30	155.45
Ankle	0.5	17.94	−12.81	5.13	−9.28	1.79	12.24
	0.8	14.37	−9.42	4.95	−7.03	2.20	9.79
	1.0	24.00	−6.42	17.59	−4.77	9.50	12.86
	1.3	21.44	−13.09	8.36	−11.46	8.63	11.19

## Data Availability

The data presented in this study are available on request from the corresponding author.
